# Variation in endoglin pathway genes is associated with preeclampsia: a case–control candidate gene association study

**DOI:** 10.1186/1471-2393-13-82

**Published:** 2013-04-01

**Authors:** Mandy J Bell, James M Roberts, Sandra A Founds, Arun Jeyabalan, Lauren Terhorst, Yvette P Conley

**Affiliations:** 1 University of Pittsburgh School of Nursing, 3500 Victoria Street, 440 Victoria Building, Pittsburgh, PA, 15261, USA; 2 Magee-Womens Research Institute and Foundation, 204 Craft Avenue, Pittsburgh, PA, 15213, USA; 3 Department of Obstetrics, Gynecology, and Reproductive Sciences, University of Pittsburgh, Pittsburgh, PA, USA; 4 Department of Epidemiology, University of Pittsburgh, Pittsburgh, PA, USA; 5 University of Pittsburgh Clinical and Translational Research, Pittsburgh, PA, USA

**Keywords:** Endoglin, Genetic association study, Preeclampsia, SNP

## Abstract

**Background:**

Preeclampsia is a hypertensive, multi-system pregnancy disorder whose pathophysiology remains unclear. Elevations in circulating soluble endoglin (sENG) and placental/blood *ENG* mRNA expression antedate the clinical onset of preeclampsia. This study investigated if endoglin (*ENG*) pathway genetic variation was also associated with the development of preeclampsia.

**Methods:**

We used a case–control candidate gene association design. Data from 355 white (181 preeclampsia cases/174 controls) and 60 black (30 preeclampsia cases/30 controls) women matched on ancestry, age, and parity were analyzed. Tagging single nucleotide polymorphisms (tSNPs) and potentially functional SNPs in *ENG*, *TGFβ1*, *TGFβR1*, *ALK1*, and *TGFβR2* were genotyped with iPLEX® and TaqMan®. Chi-square or Fisher’s exact tests were used to conduct allele/genotype/haplotype tests in white/black subgroups separately. Odds ratios were computed with binary logistic regression for tSNPs with significant genotype tests.

**Results:**

Of the 49 SNPs evaluated, variation in two *ENG* tSNPs (rs11792480, rs10121110) and one *TGFβR2* tSNP (rs6550005) was associated with preeclampsia in white women (*P* <0.05, each). In black women, variation in two *TGFβ1* tSNPs (rs4803455, rs4803457), one *TGFβR1* tSNP (rs10739778), and three *TGFβR2* tSNPs (rs6550005, rs1346907, rs877572) was associated with preeclampsia (*P* <0.05, each). Further evaluation of *ENG* tSNP rs10121110 revealed that white women inheriting the AA genotype were 2.29 times more likely to develop preeclampsia compared to the GG genotype (*P* = 0.008, [99% CI: 1.02 to 5.13]). For black women, similar evaluation of *TGFβ1* tSNP rs4803457 revealed women inheriting the CT genotype were 7.44 times more likely to develop preeclampsia than those with the CC genotype (*P* = 0.005, [99% CI: 1.19 to 46.41]).

**Conclusions:**

*ENG* pathway genetic variation is associated with preeclampsia. Different *ENG* pathway genes may be involved in preeclampsia development among white and black women. Additional studies are needed to validate these findings and to determine if genetic variation in *ENG* pathway genes impacts ENG and sENG levels in preeclampsia.

## Background

Preeclampsia is a multi-system disorder of pregnancy that complicates 3-5% of pregnancies [1] and is diagnosed by new onset hypertension and proteinuria after 20 weeks’ gestation [2,3]. The heterogeneous nature of preeclampsia suggests that multiple mechanisms lead to its development. Research has identified endoglin (ENG) as a mechanism that may contribute to preeclampsia in some women.

ENG is a trans-membrane glycoprotein that serves as a co-receptor of the transforming growth factor beta (TGFβ) signaling system [4]. It is expressed on vascular endothelial cells [5], synctyiotrophoblasts, and invasive cytotrophoblasts of cell columns [6]. ENG is involved in maintenance of vascular tone through regulation of nitric oxide dependent vasodilatation [7,8], and likely contributes to regulation of placental implantation and spiral artery remodeling during pregnancy [9,10]. Inhibition of *ENG* translation in first trimester human villous explants [9] or a human extravillous tropholast cell line [10] improves the invasive capacity of extravillous trophoblasts. Invasion of extravillous trophoblasts is proposed to be vital to uterine spiral artery remodeling, which increases placental perfusion during pregnancy [9,10].

Because systemic endothelial dysfunction and shallow placental implantation/spiral artery remodeling represent hallmark abnormalities of preeclampsia [11], ENG’s potential role has been investigated. In multiple studies, *ENG* gene expression (mRNA) is increased in placenta and/or cellular/non-cellular components of blood throughout pregnancy in women who develop preeclampsia [12-18]. Soluble endoglin (sENG), which is released into circulation after cleavage of trans-membrane ENG by matrix metalloproteinase-14 (MMP-14) [19], is also elevated in preeclampsia [20]. Our study proposed to test the role of ENG in preeclampsia by assessing if *ENG* pathway genetic variations are associated with preeclampsia.

## Methods

### Study population

Subjects were from the Prenatal Exposures and Preeclampsia Prevention (PEPP) study. Conducted at Magee-Womens Hospital of UPMC (Pittsburgh, PA), PEPP examines factors predisposing women to preeclampsia via two recruitment approaches. Women ages 14-44 were enrolled during early pregnancy (≤ 20 weeks’ gestation) and followed through delivery/postpartum while other women were enrolled at the labor/delivery unit due to suspected preeclampsia. Women with a history of chronic renal disease, hypertension, diabetes, or other disorders increasing risk of preeclampsia were excluded. Genomic DNA was extracted from peripherally collected venous blood samples. The University of Pittsburgh and Magee-Womens Hospital Institutional Review Board approved all aspects of PEPP and this study. We excluded subjects not consenting to genetic evaluation and subjects without a stored genetic sample.

### Phenotype classifications

Preeclampsia was defined as gestational hypertension and blood pressure increase, proteinuria, and hyperuricemia. These criteria were reviewed by a panel of clinicians/researchers to determine preeclampsia diagnosis. The average of the last five blood pressures taken in the hospital prior to therapeutic intervention was compared to average blood pressure prior to 20 weeks’ gestation to establish the presence/absence of a relevant increase of blood pressure. Gestational hypertension was defined as a blood pressure ≥ 140 mmHg systolic and/or 90 mmHg diastolic AND an increase of blood pressure > 30 mmHg systolic and/or 15 mmHg diastolic after 20 weeks’ gestation. Proteinuria was defined as ≥ 300 mg/24 hours, ≥ 0.3 protein/creatinine ratio, ≥ 2+ on a random urine specimen, or ≥ 1+ on a catheterized urine specimen. Hyperuricemia was a serum uric acid concentration > 1 standard deviation from normal for gestational age [21]. Severe preeclampsia was preeclampsia plus ≥ 1 of the following: (a) systolic blood pressure ≥ 160 mmHg, (b) diastolic blood pressure ≥ 110 mmHg, (c) proteinuria ≥ 5 grams/24 hours, (d) elevated liver enzymes, or (e) platelet count ≤ 100,000. Hemolysis, elevated liver enzymes, and low platelets in subjects with preeclampsia indicated HELLP syndrome. The case group included PEPP subjects diagnosed with preeclampsia, severe preeclampsia, or HELLP syndrome.

Women with normal medical histories (e.g., without chronic renal disease, hypertension, diabetes) that did not meet criteria for preeclampsia, severe preeclampsia, or HELLP syndrome were designated as healthy controls. Controls delivering prematurely (< 37 weeks’ gestation at delivery) were excluded from our analysis. A total of 215 controls were 1:1 frequency matched to 215 cases on ancestry (self-reported race), age, and parity. Data on 355 white (181 cases/174 controls) and 60 black (30 cases/30 controls) women were analyzed. In the white case group, 161 (89.0%) subjects were diagnosed with PE, 19 (10.5%) subjects were diagnosed with severe PE, and 1 (0.6%) subject was diagnosed with HELLP syndrome. In the black case group, 27 (90%) subjects were diagnosed with PE, 2 (6.7%) subjects were diagnosed with severe PE, and 1 (3.3%) subject was diagnosed with HELLP syndrome.

### Polymorphism selection

Genetic variability of the candidate genes and their regulatory regions was evaluated with tagging single nucleotide polymorphisms (tSNPs) selected from HapMap (HapMap Data Phase III/Rel#2, Feb09, on NCBI B36 assembly, dbSNP b126). Selection criteria of tSNPs included: minor allele frequency ≥20%, R^2^ cutoff = 0.8, and CEU ancestry. Forty-seven tSNPs were identified. These tSNPs and two potentially functional SNPs identified in the literature were studied. The UCSC Genome Browser [22] and Database of Single Nucleotide Polymorphisms were used to identify SNP nucleotide positions/genomic locations.

### Genotyping methods

The 49 SNPs were genotyped with the iPLEX® Gold-SNP Genotyping assay (Sequenom® Inc, San Diego, CA) (Table 1). Five SNPs (rs1800468, rs10739778, rs6809777, rs3087465, rs8179181) not meeting data quality criteria (call rate < 86% or multi-allelic >2 alleles) with iPLEX® were genotyped by TaqMan® allelic discrimination (Applied Biosystems®).

**Table 1:** tSNPS examined in iPLEX® assays

**Assay 1**		**Assay 2**	
**Gene**	**rs numbers**	**Gene**	**rs numbers**
Endoglin (*ENG)*	rs10987746, rs10819309, rs10760505, rs11792480, rs10121110	*TGFβR2*	rs2043136, rs13075948, rs1346907, rs3773652, rs4955212, rs1155708, rs3773640, rs1036097, rs2082224, rs876688, rs744751, rs1078985, rs17025785, rs877572, rs5020833, rs6809777, rs6802220, rs9843942, rs6550005, rs3773644, rs3773645, rs13083813, rs12487185, rs13086588, rs6792117, rs3773663, rs4522809, rs11129420, rs995435, rs11924422
Transforming growth factor beta 1 (*TGFβ1*)	rs4803455, rs1800469, rs4803457, rs8179181, rs1800468, rs11466314		
Transforming growth factor beta receptor 1 (*TGFβR1*)	rs6478974, rs420549, rs10739778		
Activin receptor like kinase 1 (*ALK1*)	rs3759178, rs11169953, rs706819		
Transforming growth factor beta receptor 2 (*TGFβR2*)	rs749794, rs3087465		

*Note.* The online Human GenoTyping Tools and MassARRAY® Assay Designer v4.0 software (http://www.mysequenom.com) were used to design the iPLEX® assays. To optimize the multiplex reactions, tSNPs were grouped into Assay 1 or Assay 2.

### Genotype data reliability, haplotype assignment, and linkage disequilibrium estimation

Genotype reliability checks included comparing expected to observed homozygosity, comparing study and dbSNP allele frequencies, evaluating genotype call rates, including blind duplicates, double calling genotypes, and checking Hardy-Weinberg Equilibrium (HWE). HWE calculations were conducted with PLINK v1.07 [23] or an online HWE calculator. *ENG* haplotypes and pair-wise linkage disequilibrium (R^2^) were estimated in non-related white and black subgroups separately using PLINK. Further black subgroup haplotype analysis was not conducted due to small sample size and haplotype frequencies.

### Statistical analysis

A power analysis was conducted with Quanto version 1.2.4 (Additional file 1).

White and black subgroups were evaluated separately. Demographic characteristics were compared between cases and controls. Continuous variables were assessed with independent samples t-tests, independent samples t-tests with unequal variances, or Mann–Whitney U tests. Categorical variables were assessed with the Mann–Whitney U test or the *χ2* test of independence. Missing pre-pregnancy body mass index values were estimated with multiple imputation.

Deviations from HWE were assessed with a *χ2* goodness-of-fit test or an exact test. For SNPs violating HWE (*P* < 0.05), HWE consistency was further assessed in cases and controls separately. We concluded that any SNP violating HWE in the entire group was due to enrichment for preeclampsia in cases and non-preeclampsia in controls and not due to genotyping error.

Associations between SNP alleles and preeclampsia status (allele test) were assessed with a *χ2* test of independence. Associations between SNP genotypes and preeclampsia status (genotype test) were tested with a *χ2* test of independence or Fisher’s exact test. SNPs with homozygote variant frequencies of < 10% in cases, controls, or both were dichotomized (homozygote wildtype; homozygote variant + heterozygote) before genotype testing. Given the exploratory nature of this study, we used a less stringent significance criterion of P < 0.05 to initially evaluate our genotype test results and identify potentially interesting hits. SNPs with significant findings (P < 0.05) on genotype tests were further analyzed with binary logistic regression (odds ratio and 99% CI). A 99% CI was selected for these analyses as a way to reduce the potential for Type 1 error due to multiple testing. Analyses were conducted with SPSS version 19 (SPSS Inc., Chicago, IL.).

The most probable *ENG* haplotypes estimated for each white subject were selected for analysis. Haplotype frequencies with < 10% in cases, controls, or both were collapsed into one category (Additional file 1: Table S1). A *χ2* test of independence was used to determine if haplotype frequency distributions differed in cases and controls. Separate pair-wise comparisons of haplotype frequency distributions were also analyzed. The association between diplotypes, which were generated from *ENG* haplotypes, and preeclampsia status was assessed with a *χ2* test of independence.

## Results

### Allele/genotype findings support association between *ENG* pathway genetic variation and preeclampsia

Tables 2 and 3 provide demographic/clinical characteristics for white and black subgroups and reveal the success of matching (ancestry, age, and parity) and the expected differences between women with preeclampsia and controls (blood pressure, gestational age at delivery, and BMI). Additional file 1: Tables S2-S5 provide descriptive information and allele/genotype test results.

**Table 2:** White subgroup demographic and clinical characteristics

**Variable**	**Cases (n = 181)**	**Controls (n = 174)**	**p-value**
Maternal age, years *(M (SD))*	28.3 (5.8)	28.4 (5.7)	0.87^a^
Gravida *(Mdn (min-max))*	1 (1-6)	1 (1-8)	0.08^b^
Nulliparous (n, %)	146 (80.7%)	139 (79.9%)	0.85^c^
Gestational age at delivery, wks *(M (SD))*	36.1 (3.2)	39.6 (1.1)	**< 0 .001**^d^
Birthweight, grams *(M (SD))*^e^	2497.5 (841.2)	3481.6 (446.3)	**< 0.001**^d^
Avg. SBP < 20 weeks’, mm Hg *(M (SD))*^f^	116.6 (9.6)	112.1 (7.5)	**< 0.001**^d^
Avg. DBP < 20 weeks’, mm Hg *(M (SD))*^f^	71.7 (7.2)	68.1 (4.9)	**< 0.001**^d^
Avg. SBP in labor, mm Hg *(M (SD))*^g^	154.8 (13.9)	120.4 (10.2)	**< 0.001**^d^
Avg. DBP in labor, mm Hg *(M (SD))*^h^	92.6 (8.0)	72.3 (7.2)	**< 0. 001**^a^
Pre-pregnancy BMI *(Mdn (min-max))*^i^	25.8 (17-46)	22.9 (16-37)	**< 0.001**^b^

Abbreviations: *(M (SD))*, mean (standard deviation); *(Mdn (min-max))*, median (minimum-maximum); wks, weeks; Avg. SBP, average systolic blood pressure; mmHg, millimeters of mercury; Avg. DBP, average diastolic blood pressure; BMI, body mass index.

^a^ Independent samples *t*-test.

^b^ Mann–Whitney U test.

^c^*χ2* test of independence.

^d^ Independent samples *t*-test with unequal variance.

^e^ Outlier removed in controls.

^f^*n* = 168 cases &*n* = 170 controls.

^g^*n* = 181 cases &*n* = 173 controls.

^h^*n* = 180 cases &*n* = 173 controls.

^i^ BMI values imputed for *n* = 22 cases, sample size *n* = 178 cases &*n* = 172 controls.

**Table 3:** Black subgroup demographic and clinical characteristics

**Variable**	**Cases (n = 30)**	**Controls (n = 30)**	**p-value**
Maternal age, years *(Mdn (min-max))*	20.0 (14-37)	20.0 (14-37)	0.99^a^
Gravida *(Mdn (min-max))*	1 (1-6)	1 (1-6)	0.38^a^
Nulliparous *(n, %)*	25 (83.3%)	25 (83.3%)	-------
Gestational age at delivery, wks *(Mdn (min-max))*	36.9 (27.4-40.0)	40.6 (37.1-42.1)	**< 0.001**^a^
Birthweight, grams *(M (SD))*	2313.9 (715.8)	3388.8 (405.3)	**< 0.001**^b^
Avg. SBP < 20 weeks’, mm Hg *(M (SD))*^c^	113.3 (9.2)	114.4 (6.8)	0.60^d^
Avg. DBP < 20 weeks’, mm Hg *(M (SD))*^c^	70.4 (6.5)	69.2 (4.2)	0.41^d^
Avg. SBP in labor, mm Hg *(M (SD))*^e^	159.9 (17.8)	120.7 (9.1)	**< 0.001**^b^
Avg. DBP in labor, mm Hg *(M (SD))*^e^	97.1 (10.4)	72.6 (7.4)	**< 0.001**^d^
Pre-pregnancy BMI *(Mdn (min-max))*^f^	23.0 (17.7-38.4)	25.8 (19.4-49.9)	0.25^a^

Abbreviations: *(Mdn (min-max))*, median (minimum-maximum); wks, weeks; *(M (SD))*, mean (standard deviation); Avg. SBP, average systolic blood pressure; mmHg, millimeters of mercury; Avg. DBP, average diastolic blood pressure; BMI, body mass index.

^a^ Mann–Whitney *U* test.

^b^ Independent samples *t*-test with unequal variances.

^c^*n* = 24 cases &*n* = 29 controls.

^d^ Independent samples *t*-test.

^e^*n* = 30 cases &*n* = 29 controls.

^f^*n* = 27 cases &*n* = 30 controls.

We examined if the proportion of alleles for each of the 49 SNPs (allele 1 or allele 2) differed in women with preeclampsia compared to controls. Using P < 0.05 to indicate statistical significance, allelic frequency distributions for two tSNPs in *ENG* (rs11792480: G/A alleles; rs10121110: A/G alleles) and one tSNP in *TGFβR2* (rs6550005: G/A alleles) were significantly different in white cases and controls. The G allele of rs11792480 (71.7% vs. 63.0%, *P* = 0.01), the A allele of rs10121110 (66.0% vs. 55.3%, *P* = 0.004), and the G allele of rs6550005 (84.0% vs. 77.6%, *P* = 0.03) were overrepresented in white cases. Allelic distributions for all SNPs in *TGFβR1*, *ALK1*, and *TGFβ1*, along with the remaining SNPs in *ENG* and *TGFβR2*, were not significantly different in the white subgroup.

The genotype for *ENG* tSNP rs10121110 (AA vs. GG vs. AG) was also significantly associated with preeclampsia (*P* = 0.02) in the white subgroup. This association was further explored with binary logistic regression, and odds ratios were generated. Because of multiple testing, we evaluated logistic regression results with *α* = 0.01 (Table 4). Women inheriting the AA genotype for rs10121110 were 2.29 times more likely to develop preeclampsia compared to women inheriting the GG genotype (*P* = 0.008, [99% CI: 1.02 to 5.13]). The genotype for *TGFβR2* tSNP rs6550005 (GG vs. AA + GA) was also significantly associated with preeclampsia in the white subgroup (*P* = 0.04), but further exploration of this association with logistic regression and a more stringent criterion for significance (P < 0.01) did not support this association (Table 4). Genotype tests for all SNPs in *TGFβR1*, *ALK1*, and *TGFβ1*, along with the remaining SNPs in *ENG* and *TGFβR2*, demonstrated no significant differences in whites.

**Table 4:** Logistic regression for tSNPs with significant genotype tests

**White Subgroup**				
**Gene/tSNP**	**Genotype Groups**	**OR**	**99% CI**	**p-value**^**a**^
*ENG:* rs10121110	AA vs. GG	2.29	1.02 - 5.13	**0.008**
	AG vs. GG	1.52	0.69 - 3.36	0.17
*TGFBR2:* rs6550005^b^	GG vs. AA + GA	1.60	0.89 - 2.87	0.04
**Black Subgroup**				
**Gene/tSNP**	**Genotype Groups**	**OR**	**99% CI**	**p-value**
*TGFβ1:* rs4803455	AA vs. CC	0.39	0.06 - 2.61	0.20
	CA vs. CC	3.04	0.56 - 16.33	0.09
*TGFβ1:*rs4803457	TT vs. CC	3.50	0.50 - 24.53	0.10
	CT vs. CC	7.44	1.19 - 46.41	**0.005**
*TGFβR1:* rs10739778	CC vs. AA	0.19	0.02 - 2.08	0.07
	AC vs. AA	0.24	0.05 - 1.09	0.02

Abbreviations: *tSNP*, tagging single nucleotide polymorphism; *OR*, odds ratio; *CI*, confidence interval.

^a^ α set at 0.01 because of multiple testing.

^b^ SNP genotypes dichotomized (homozygote wildtype, homozygote variant + heterozygote) due to small homozygote variant frequencies in either cases, controls, or both.

In the black subgroup, allelic frequency distributions for one tSNP in *TGFβR1* (rs10739778: A/C alleles) and three tSNPs in *TGFβR2* (rs6550005: G/A alleles; rs1346907: C/T alleles; rs877572: G/C alleles) were significantly different in cases and controls. The A allele of rs10739778 (79.3% vs. 56.7%, *P* = 0.008), the A allele of rs6550005 (46.7% vs. 26.7%, *P* = 0.02), the C allele of rs1346907 (70.0% vs. 63.3%, *P* = 0.04), and the G allele of rs877572 (70.0% vs. 61.7%, *P* = .03) were overrepresented in black cases. Allelic distributions for all SNPs in *ALK1*, *TGFβ1*, and *ENG*, along with the remaining SNPs in *TGFβR1*, *TGFβR2*, were not significantly different in the black subgroup.

The genotype for *TFGβ1* tSNP rs4803455 (CC vs. AA vs. CA) was significantly associated with preeclampsia (*P* = 0.01) in the black subgroup. However, evaluation of the association by binary logistic regression did not support this association at α = 0.01 (Table 4). *TGFβ1* tSNP rs4803457 genotype (CC vs. TT vs. CT) was also significantly associated with preeclampsia in the black subgroup (*P* = 0.01). Further analysis of rs4803457 revealed that women inheriting the CT genotype were 7.44 times more likely to develop preeclampsia compared to women inheriting the CC genotype (*P* = 0.005, [99% CI: 1.19 to 46.41]). Lastly, the genotype for *TGFβR1* tSNP rs10739778 (AA vs. CC vs. AC) was significantly associated with preeclampsia in the black subgroup (*P* = 0.03). However, further exploration with binary logistic regression did not support this association (Table 4). Genotype tests for all SNPs in *ALK1*, *ENG*, and *TGFβR2*, along with the remaining SNPs in *TGFβR1*, and *TGFβ1*, demonstrated no significant differences in the black subgroup.

### *ENG* haplotype TACGA associated with preeclampsia in white subgroup

We estimated linkage disequilibrium and haplotypes for *ENG* in whites. Pairwise R^2^ values ranged from 0.007-0.64. For rs10121110 and rs11792480, which were both significantly associated with preeclampsia and are separated by about 4000 bases, R^2^ = 0.29. These results validate our tSNP selection criteria and indicate that haploblocks tagged by rs10121110 and rs11792480 are independently associated with preeclampsia.

Nineteen possible haplotypes were estimated across the *ENG* tSNPs (rs10987746, rs10819309, rs10760505, rs11792480, rs10121110). Seventeen of the haplotypes were present in whites (Additional file 1: Table S1). *ENG* haplotype distributions (CGTGA, TACAG, TACGA, and combined) were significantly different in cases and controls (*χ2*(3) = 8.26, *P* = 0.04). The TACGA allele, which contains the risk alleles from our significantly associated tSNPS, was over-represented in cases (*χ2*(1) = 5.23, *P* = 0.02) when compared to the other alleles combined. We also observed a 6.1% difference in TACGA frequency among whites and blacks (19.7% vs.13.6%). Diplotype analysis in whites found no significant differences in *ENG* diplotypes among cases and controls (*χ2*(4) = 7.28, *P* = 0.12).

## Discussion

This study begins to examine the association between *ENG* pathway genetic variation and preeclampsia. In our white sample, variation in *ENG* and *TGFβR2* appears to be associated with preeclampsia while variation in *TGFβ1* (excluding rs8179181- not genotyped), *TGFβR1*, and *ALK1* was not associated with preeclampsia. In our smaller black sample, pathway associations also appeared, but were different. As with whites, variation in *TGFβR2* was associated with preeclampsia, but variation in *ENG* was not. In blacks, but not whites, pathway variation was suggested for *TGFβ1* and *TGFβR1*. As with whites, variants in *ALK1* were not associated with preeclampsia. These results suggest that *ENG* pathway genetic variation is associated with preeclampsia, with different pathway genes contributing to preeclampsia development in white and black women.

In this study we used a very strict definition of preeclampsia that included both incremental and absolute increases in blood pressure, as well as hyperuricemia. We believe such rigor is necessary for genetic studies of preeclampsia, but this approach limited sample sizes. Further, because of multiple comparisons, we explored significant genotype findings (P < 0.05) with binary logistic regression using a more stringent level of significance (α = 0.01), which again limits our definitive findings. Nonetheless, we believe that using this strategy of examining genotypic variation in pathway analysis to support the role of a potential pathogenic factor provides useful insights to guide further studies of specific pathway variation.

**Figure 1:**
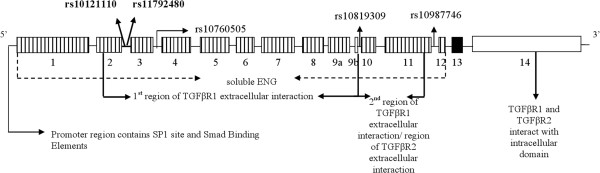
**Endoglin gene structure.***ENG* extracellular domain exons (vertical lines), transmembrane domain exon (black), and the intracellular domain exon (white). tSNPs with significant allele &/or genotype tests are bolded. The following resources were utilized to create the figure [22,24-27].

Are the pathway associations we have demonstrated biologically plausible? Although we do not know the functional consequences of these associations, several explanations could account for the association between *ENG* (rs10121110 and rs11792480) and *TGFβR2* (rs6550005) genetic variation and preeclampsia in white women. Intronically located between the second and third exons, rs10121110 tags a genomic region that includes *ENG*’s promoter (Figure 1). Given rs10121110’s location, this tSNP potentially identifies a correlated promoter variant that has the ability to impact transcription factor access/binding (e.g., SP1 transcription factor, SMAD binding elements) [24] and subsequent transcription/translation of *ENG*.

Knockdown of *ENG* in a human extravillous trophoblast cell line with short hairpin RNA specific for *ENG*[10] or knockdown of first trimester human trophoblast villous explants with antisense endoglin nucleotides [9] improves extravillous trophoblast invasive capacity, which is essential to uterine spiral artery remodeling in pregnancy [9,10]. In preeclampsia, placental concentrations of *ENG* mRNA are elevated throughout pregnancy [12,16-18] and spiral artery remodeling is shallow [11]. Therefore, a variant within *ENG*’s promoter could increase expression of placental *ENG*, which could inhibit extravillous trophoblast invasion of the spiral arteries, leading to shallow implantation and reduced placental perfusion. In a setting with increased concentration of membrane-bound ENG receptors, mass action predicts that more will be cleaved by MMP-14, resulting in increased sENG, which is found in women with preeclampsia and has been suggested to cause endothelial dysfunction [18]. Studies examining *ENG*’s promoter, its transcription factors, and MMP-14 are needed to better understand mechanisms that drive observed differences in ENG and sENG.

*ENG* tSNP rs10121110 is also located between exons coding for ENG’s extracellular domain, as is rs11792480, the other tSNP associated with preeclampsia in whites (Figure 1). As part of TGFβ1’s signaling cascade, TGFβR1 interacts with amino acid residues 26-437 of ENG’s extracellular domain [25]. Only through interaction of ENG and type 1/2 receptors can ENG gain access to TGFβ1 [25]. Genetic variation within exons that code for the extracellular domain could therefore influence ENG’s ability to interact with TGFβR1, affecting ENG’s access to TGFβ1 and the transmission of TGFβ1 signals. Because TGFβ1 induces *ENG* expression [10] and stimulates *ENG* promoter activity [28], genetic variation that affects the degree of TGFβ1 transmission may also explain differences in *ENG* expression (mRNA) between women with/without preeclampsia. Studies examining genetic regions tagged by rs10121110 and rs11792480 may provide insight into ENG’s involvement in preeclampsia.

*TGFβR2* tSNP rs6550005, which was associated with preeclampsia in both groups, is intronically located between the first two exons that lie adjacent to the *TGFβR2* promoter. As a result, rs6550005 may tag a promoter variant that influences *TGFβR2* transcription and translation. Because ENG only binds TGFβ1 ligand in the presence of type 1/2 signaling receptors [25], alteration in *TGFβR2* transcription/translation could impact the number of TGFβR2 receptors available for ENG interaction and transmission of TGFβ1 ligand signaling. Furthermore, this association in whites and blacks potentially indicates that there is also one component of the ENG pathway that similarly contributes to PE development regardless of ancestry, but because the minor allele frequencies differed in whites and blacks (0.192 vs. 0.367), we did not combine the data from these two groups.

Our exploratory examination in black women revealed that different genes from different components of the *ENG* pathway (*TGFβ1* and *TGFβR1*, but not *ENG*) were associated with preeclampsia compared to white women. These results suggest that the pathway’s involvement in preeclampsia may differ in blacks and whites. Interestingly, in a study comparing TGFβ1 mRNA and protein levels in nonpregnant black and white hypertensive subjects, TGFβ1 protein levels were significantly higher in blacks compared to whites (*P* < 0.001) [29].

One additional study has investigated the association between *ENG* and preeclampsia in white and black women using a pre-designed IBCv2 array (Illumina Inc, San Diego, CA) [30]. Unlike the significant associations found between *ENG* and preeclampsia in our white subgroup, their study failed to find significant associations in a much smaller white subgroup that was likely underpowered. Consistency in our findings from allele, genotype, and haplotype tests of *ENG* increases our confidence in our findings. In both studies, associations between *ENG* and preeclampsia were non-significant in blacks.

In addition to sample size, there are other limitations to our study. Multiallelic tSNPs rs8179181 (*TGFβ1*) and rs3087465 (*TGFβR2*) could not be genotyped despite multiple attempts with iPLEX® and TaqMan®, which limits our ability to fully evaluate *TGFβ1* and *TGFβR2* genetic variability. Our black subgroup was likely underpowered. This was not an issue in the white sample, which generated a power ranging from 0.898 to 0.999 (Additional file 1). Additionally, we selected tSNPs for CEU ancestry, which may have resulted in decreased informativeness in blacks since haploblocks tagged by tSNPs selected for CEU ancestry may differ from haploblocks tagged by tSNPs selected for African ancestry. Finally, we used self-reported race to match controls to cases on ancestry. Because this may not reliably account for population admixture, the use of ancestral informative markers in future studies represents a more robust approach that statistically accounts for population admixture.

## Conclusions

In summary, our study demonstrated that *ENG* pathway genetic variation is associated with preeclampsia in white and black women. Our results further suggest that the pathway’s involvement in preeclampsia differs in whites and blacks, with *ENG* and *TGFBR2* being associated in whites and *TGFβ1*, *TGFβR1*, and *TGFβR2* being associated in blacks. Validation of results is needed to confirm these preliminary findings. Moreover, the *ENG* pathway tSNPs found to be significantly associated with preeclampsia likely represent surrogate markers, which tag genomic regions that contain causal variants. Focused examination of genomic regions (e.g., *ENG* promoter sequencing) tagged by these SNPs will further improve understanding of the ENG pathway’s role in preeclampsia.

## Competing interests

The authors declare that they have no competing interests.

## Authors’ contributions

MB participated in the conception and design of research, acquisition of data, analysis and interpretation of data, and drafting of the manuscript. JR participated in the acquisition of data/samples, analysis and interpretation of data, and critical revision of the manuscript for important intellectual content. SF participated in the interpretation of data and critical revision of the manuscript for important intellectual content. AJ participated in the recruitment of subjects, acquisition of data, and critical revision of the manuscript for important intellectual content. LT participated in the analysis and interpretation of data and critical revision of the manuscript for important intellectual content. YP participated in conception and design of research, interpretation of data, drafting the manuscript, and critical revision of the manuscript for important intellectual content. All authors read and approved the final manuscript.

## Pre-publication history

The pre-publication history for this paper can be accessed here:

http://www.biomedcentral.com/1471-2393/13/82/prepub

## Supplementary Material

Additional file 1 Variation in endoglin pathway genes is associated with preeclampsia: A case-control candidate gene association study. Click here for file
