# Supplementary material for: Variation in endoglin pathway genes is associated with preeclampsia: a case–control candidate gene association study
Source: BMC Pregnancy Childbirth. 2013 Apr 1;13:82. doi: 10.1186/1471-2393-13-82 (PMC3651360; doi:10.1186/1471-2393-13-82)
Supplement: Additional file 1 — Variation in endoglin pathway genes is associated with preeclampsia: A case-control candidate gene association study. [file 1471-2393-13-82-S1.docx]

SUPPLEMENTARY MATERIAL

**Variation in endoglin pathway genes is associated with preeclampsia: A case-control candidate gene association study**

Mandy J Bell^1,2^*, James M Roberts^2,3,4,5^, Sandra A Founds^1,2^, Arun Jeyabalan^2,3^, Lauren Terhorst^1^, Yvette P. Conley^1^

^1^University of Pittsburgh School of Nursing

3500 Victoria

440 Victoria Building

Pittsburgh, PA 15261

^2^Magee-Womens Research Institute and Foundation

204 Craft Avenue

Pittsburgh, PA 15213

^3^University of Pittsburgh Department of Obstetrics, Gynecology, and Reproductive Sciences (Pittsburgh, PA)

^4^University of Pittsburgh Department of Epidemiology (Pittsburgh, PA)

^5^University of Pittsburgh Clinical and Translational Research (Pittsburgh, PA)

*Corresponding author

**Methods: Power Analysis**

Quanto version 1.2.4 was used to conduct a power analysis for this genetic association study. Information for the following program parameters were entered: (1) SNP minor allele frequency: 0.2 - 0.5; (2) no environmental influence; (3) additive gene effect due to preeclampsia’s multi-factorial nature; (4) 1:1 case/control matching based on study design; (5) population risk of 5% [1]; (6) heritability factor range 0.31-0.54 [2-3]; (7) α =.05; and (8) two-sided test. A sample of 225 cases resulted in statistical power ranging from 0.9496-0.9999. A post hoc power analysis using Quanto version 1.2.4 revealed that a sample of 181 controls matched to 181 cases resulted in a power ranging from .8979 to .9990, using the same parameters noted above. Application of a more stringent alpha level (.01) resulted in a power ranging from .7434 to 0.9934 in a sample of 181 controls matched to 181 cases.

1. Roberts JM, Cooper DW: **Pathogenesis and genetics of pre-eclampsia.** *Lancet* 2001, **357**:53-56.
2. Nilsson E, Salonen Ros H, Cnattinguis S, Lichtenstein P: **The importance of genetic and environmental effects for pre-eclampsia and gestational hypertension: A family study.** *Br J Obstet Gynaecol* 2004, **111**:200-206.
3. Salonen Ros H, Lichtenstein P, Lipworth L, Cnattingius S: **Genetic effects on the liability of developing pre-eclampsia and gestational hypertension.** *AM J Med Genet* 2000, **91**:256-260.

**Table S1.** ***ENG* haplotype frequencies in white cases & controls**

| Haplotype | Cases (*n* = 180 subjects)  Allele Counts  N (%) | Controls (*n* = 170 subjects)  Allele Counts  N (%) |
| --- | --- | --- |
| CGTGA | 94 (26.1%) | 79 (23.2%) |
| TACAG | 51 (14.2%) | 51 (15.0%) |
| TACGA | 83 (23.1%) | 55 (16.2%) |
| Combined: CGCAA, CGCAG, CGCGG, CGTAA, CGTAG, CGTGG, TACGG, TGCAG, TGCGA, CGCGA, TACAA, TGCGG, CATAA, TGTGG | 132 (36.7%) | 155 (45.6%) |

tSNP order for haplotype assignment was rs10987746, rs10819309, rs10760505, rs11792480, rs10121110. Allele frequencies based on analysis of the most probable haplotypes for each subject. Haplotypes CATAG and TATAG (not listed above) did not represent the most probable haplotype for any of the subjects and were not included in any analyses.

**Table S2. tSNP and functional SNP information in white subgroup (N=355)**

| Gene-Chromosome  tSNP | Wildtype Allele/ Nucleotide #/ Variant Allele^a^ | *n for each SNP* | Study MAF | HapMap MAF | HWE^b^ |
| --- | --- | --- | --- | --- | --- |
| *ALK5 (TGFBR1)-Chr9* |  |  |  |  |  |
| rs6478974 | T/101874403/A | 349 | A = .483 | A = .469 | p = .434 |
| rs10739778 | A/101875789/C | 352 | C = .314 | C = .292 | **p = .040** |
| rs420549 | G/101914873/C | 350 | C = .169 | C = .204 | p = .433 |
| *ALK1-Chr12* |  |  |  |  |  |
| rs3759178 | T/52299259/G | 350 | G = .369 | G = .398 | p = .917 |
| rs11169953 | C/52304399/T | 350 | T = .351 | T = .250 | p = .958 |
| rs706819 | G/52315923/A | 350 | A = .226 | A = .286 | p = .799 |
| *TGFB1-Chr19* |  |  |  |  |  |
| rs8179181 | --/41838206/-- | ---- | ---- | ---- | ---- |
| rs4803455 | C/41851509/A | 350 | A = .487 | A = .496 | p = .840 |
| rs11466314 | G/41860236/A | 350 | A = .000 | A = .000 | ---- |
| rs1800469 | C/41860296/T | 350 | T = .301 | T = .288 | p = .286 |
| rs1800468 | C/41860587/T | 353 | T = .086 | T = .050 | p = .312 |
| rs4803457 | C/41861359/T | 350 | T = .384 | T = .381 | p = .601 |
| *ENG-Chr9* |  |  |  |  |  |
| rs10987746 | T/130580093/C | 349 | C = .493 | C = .415 | p = .869 |
| rs10819309 | G/130581723/A | 349 | A =.367 | A = .473 | p = .99 |
| rs10760505 | C/130589853/T | 348 | T = .386 | T = .341 | p = .495 |
| rs11792480 | G/130598125/A | 350 | A = .326 | A = .353 | **p = .008** |
| rs10121110 | A/130602408/G | 348 | G = .392 | G = .412 | p = .057 |
| *TGFBR2-Chr3* |  |  |  |  |  |
| rs3087465 | --/30647160/-- | ---- | ---- | ---- | ---- |
| rs6550005 | G/30650064/A | 355 | A = .192 | A = .243 | p = .088 |
| rs11129420 | A/30658541/T | 355 | T = .475 | A = .487 | p = .671 |
| rs6802220 | G/30659652/A | 355 | A = .414 | A = .363 | p = .199 |
| rs17025785 | T/30667425/C | 355 | C = .334 | C = .330 | p = .708 |
| rs4522809 | C/30668684/T | 355 | T = 0.499 | C = .478 | p = .708 |
| rs4955212 | C/30669358/T | 351 | T = .268 | T = .252 | p = .752 |
| rs5020833 | C/30670425/G | 355 | G = .304 | G = .296 | p = .823 |
| rs6809777 | C/30672362/T | 355 | T = .261 | T = .270 | p = .104 |
| rs12487185 | A/30677269/G | 355 | G = .283 | G = .323 | p = .146 |
| rs11924422 | A/30677484/C | 355 | C = .431 | C = .456 | p = .655 |
| rs13083813 | T/30679558/A | 354 | A = .377 | A = .398 | p = .888 |
| rs13075948 | C/30683506/T | 355 | T = .275 | T = .265 | p > .999 |
| rs1155708 | G/30686740/A | 354 | A = .322 | A = .336 | p = .424 |
| rs13086588 | T/30688757/G | 355 | G = .314 | G = .332 | p = .141 |
| rs2082224 | G/30689755/A | 355 | A = .235 | A = .235 | p = .920 |
| rs1078985 | T/30690911/C | 349 | C = .256 | C = .332 | p = .764 |
| rs1036097 | G/30693643/A | 355 | A = .472 | A = .412 | p = .671 |
| rs995435 | C/30700922/T | 348 | T = .263 | T = .257 | p = .791 |
| rs6792117 | A/30704007/G | 353 | G = .482 | A = .451 | p = .689 |
| rs749794 | T/30708432/C | 350 | C = .319 | C = .332 | p = .532 |
| rs3773640 | A/30709511/T | 355 | T = .242 | T = .270 | p > .999 |
| rs3773644 | C/30712344/T | 355 | T = .380 | T = .438 | p = .450 |
| rs3773645 | C/30712460/G | 354 | G = .301 | G = .367 | p = .308 |
| rs3773652 | A/30718942/G | 355 | G = .480 | G = .469 | **p = .032** |
| rs2043136 | T/30720304/C | 355 | C = .268 | C = .239 | p = .484 |
| rs1346907 | C/30723470/T | 355 | T = .473 | T = .451 | **p = .001** |
| rs876688 | G/30725776/A | 354 | A = .340 | A = .376 | p = .238 |
| rs877572 | G/30726432/C | 355 | C = .466 | C = .460 | **p = .021** |
| rs9843942 | G/30729636/A | 354 | A = .380 | A = .375 | p = .842 |
| rs3773663 | G/30730872/A | 355 | A = .396 | A = .429 | p = .752 |
| rs744751 | C/30735937/T | 355 | T = .265 | T = .363 | p = .396 |

Abbreviations: MAF, mean allele frequency; HWE, hardy weinberg equilibrium

^a^ wildtype and variant alleles based on study sample

^b^ *χ2* goodness-of-fit test or exact test (significant results bolded)

---- = not analyzed

Of the 46 SNPs included in the analysis, five tSNPs violated HWE (p < .05) in the white subgroup. One tSNP was located in TGFβR1 (rs10739778), one tSNP was located in ENG (rs11792480), and three tSNPs were located in TGFβR2 (rs3773652, rs1346907, rs877572). Separate evaluation of HWE in cases and controls revealed that rs10739778 was in HWE in controls (p = .846), rs11792480 was in HWE in cases (p = .193), rs3773652 was in HWE in controls and cases separately (p = .069 & p = .238), rs1346907 was in HWE in controls (p = .098), and rs877572 was in HWE in controls (p = .315).

**Table S3. tSNP and functional SNP information in black subgroup (N=60)**

| Gene-Chromosome  tSNP | Wildtype Allele/ Nucleotide #/ Variant Allele^a^ | *n for each SNP* | Study MAF | HapMap MAF | HWE^b^ |
| --- | --- | --- | --- | --- | --- |
| *ALK5 (TGFBR1)-Chr9* |  |  |  |  |  |
| rs6478974 | T/101874403/A | 59 | A = .237 | A = .075 | p = .276 |
| rs10739778 | A/101875789/C | 59 | C = .322 | C = .339 | p = .597 |
| rs420549 | G/101914873/C | 59 | C = .119 | C = .106 | p = .582 |
| *ALK1-Chr12* |  |  |  |  |  |
| rs3759178 | T/52299259/G | 59 | G = .398 | T = .491 | p = .146 |
| rs11169953 | C/52304399/T | 59 | T = .466 | T = .482 | **p = .028** |
| rs706819 | G/52315923/A | 59 | A = .297 | A = .236 | p = .899 |
| *TGFB1-Chr19* |  |  |  |  |  |
| rs8179181 | --/41838206/-- | ---- | ---- | ---- | ---- |
| rs4803455 | C/41851509/A | 59 | A = .492 | C = .397 | p = .157 |
| rs11466314 | G/41860236/A | 59 | A = .051 | A = .025 | ---- |
| rs1800469 | C/41860296/T | 59 | T = .271 | T = .208 | p = .741 |
| rs1800468 | C/41860587/T | 60 | T = .050 | T = .033 | p > .999 |
| rs4803457 | C/41861359/T | 59 | T = .483 | T = .420 | p = .237 |
| *ENG-Chr9* |  |  |  |  |  |
| rs10987746 | T/130580093/C | 59 | C = .458 | C = .438 | p = .371 |
| rs10819309 | G/130581723/A | 59 | A = .322 | A = .195 | p = .249 |
| rs10760505 | C/130589853/T | 59 | T = .119 | T = .058 | p = .169 |
| rs11792480 | G/130598125/A | 59 | A = .119 | A = .004 | p = .169 |
| rs10121110 | G/130602408/A | 59 | A = .381 | A = .310 | p = .823 |
| *TGFBR2-Chr3* |  |  |  |  |  |
| rs3087465 | --/30647160/-- | ---- | ---- | ---- | ---- |
| rs6550005 | G/30650064/A | 60 | A = .367 | G = .491 | p = .294 |
| rs11129420 | A/30658541/T | 60 | T = .292 | T = .097 | p = .572 |
| rs6802220 | A/30659652/G | 60 | G = .358 | G = .243 | p = .484 |
| rs17025785 | T/30667425/C | 60 | C = .300 | C = .283 | p = .380 |
| rs4522809 | T/30668684/C | 60 | C = .475 | C = .411 | p = .069 |
| rs4955212 | C/30669358/T | 60 | T = .208 | T = .208 | p = .429 |
| rs5020833 | C/30670425/G | 60 | G = .292 | G = .142 | p = .233 |
| rs6809777 | C/30672362/T | 60 | T = .225 | T = .204 | p = .147 |
| rs12487185 | A/30677269/G | 60 | G = .200 | G = .088 | **p = .007** |
| rs11924422 | A/30677484/C | 60 | C = .367 | C = .381 | p = .554 |
| rs13083813 | T/30679558/A | 60 | A = .225 | A = .075 | p = .261 |
| rs13075948 | C/30683506/T | 60 | T = .192 | T = .155 | p > .999 |
| rs1155708 | G/30686740/A | 60 | A = .392 | A = .358 | p = .913 |
| rs13086588 | T/30688757/G | 60 | G = .375 | G = .319 | p = .403 |
| rs2082224 | G/30689755/A | 60 | A = .267 | A = .243 | p > .999 |
| rs1078985 | T/30690911/C | 59 | C = .169 | C = .221 | **p = .047** |
| rs1036097 | G/30693643/A | 60 | A = .333 | A = .292 | p = .856 |
| rs995435 | C/30700922/T | 59 | T = .356 | T = .429 | p = .390 |
| rs6792117 | A/30704007/G | 60 | G = .383 | G = .363 | p = .655 |
| rs749794 | T/30708432/C | 59 | C = .381 | C = .451 | p = .442 |
| rs3773640 | A/30709511/T | 60 | T = .250 | T = .248 | p = .487 |
| rs3773644 | C/30712344/T | 60 | T = .325 | T = .332 | p = .856 |
| rs3773645 | C/30712460/G | 60 | G = .225 | G = .111 | p = .055 |
| rs3773652 | G/30718942/A | 60 | A = .467 | A = .292 | p = .277 |
| rs2043136 | T/30720304/C | 60 | C = .225 | C = .279 | p = .712 |
| rs1346907 | C/30723470/T | 60 | T = .283 | T = .221 | p > .001 |
| rs876688 | G/30725776/A | 60 | A = .433 | A = .429 | p = .517 |
| rs877572 | G/30726432/C | 60 | C = .292 | C = .246 | p = .549 |
| rs9843942 | G/30729636/A | 60 | A = .467 | G = .385 | p = .310 |
| rs3773663 | G/30730872/A | 60 | A = .450 | A = .385 | p = .938 |
| rs744751 | C/30735937/T | 60 | T = .125 | T = .058 | p > .001 |

Abbreviations: MAF, mean allele frequency; HWE, hardy weinberg equilibrium;

^a^ wildtype and variant alleles based on study sample

^b^ *χ2* goodness-of-fit test or exact test (significant results bolded)

---- = not analyzed

Of the 46 SNPs analyzed, 3 tSNPs violated HWE (p < .05) in the black subgroup. One tSNP was located in ALK1 (rs11169953) and two tSNPs were located in TGFβR2 (rs12487185 and rs1078985). Separate evaluation of HWE in cases and controls revealed that rs11169953 was in HWE in cases and controls separately (p = .119 & p = .145), rs12487185 was in HWE in cases (p = .099), and rs1078985 was in HWE in cases and controls separately (p = .155 & p = .165).

**Table S4. Results of association analysis in white subgroup (Total N=355)**

| Gene  SNP | Allele Counts (%) Cases | Allele Counts (%) Controls | Total Alleles (*n)* | Allele Test^a^ | Genotype Counts (%) Cases | Genotype Counts (%) Controls | Total Genotypes (*n*) | Genotype Test^b^ |
| --- | --- | --- | --- | --- | --- | --- | --- | --- |
| *ALK5 (TGFBR1)* |  |  |  |  |  |  |  |  |
| rs6478974 | T: 185 (51.7%)  A: 173(48.3%) | T: 176 (51.8%)  A: 164 (48.2%) | 698 | p > .999 | TT: 50 (27.9%)  AA: 44 (24.6%)  TA: 85 (47.5%) | TT: 47 (27.6%)  AA: 41 (24.1%)  TA: 82 (48.2%) | 349 | p = .990 |
| rs10739778^c^ | A: 244 (68.2%)  C: 114 (31.8%) | A: 239 (69.1%)  C: 107 (30.9%) | 704 | p = .791 | AA: 92 (51.4%)  CC: 27 (15.1%)  AC: 60 (33.5%) | AA: 82 (47.4%)  CC: 16 (9.2%)  AC: 75 (43.4%) | 352 | p = .453 |
| rs420549^c^ | G: 296 (82.2%)  C: 64 (17.8%) | G: 286 (84.1%)  C: 54 (15.9%) | 700 | p = .502 | GG: 125 (69.4%)  CC: 9 (5.0%)  GC: 46 (25.6%) | GG: 119 (70.0%)  CC: 3 (1.8%)  GC: 48 (28.2%) | 350 | p = .910 |
| *ALK1* |  |  |  |  |  |  |  |  |
| rs3759178 | T: 235 (65.3%)  G: 125 (34.7%) | T: 207 (60.9%)  G: 133 (39.1%) | 700 | p = .229 | TT: 75 (41.7%)  GG: 20 (11.1%)  GT: 85 (47.2%) | TT: 65 (38.2%)  GG: 28 (16.5%)  GT: 77 (45.3%) | 350 | p = .340 |
| rs11169953 | C: 228 (63.3%)  T: 132 (36.7%) | C: 226 (66.5%)  T: 114 (33.5%) | 700 | p = .383 | CC: 72 (40.0%)  TT: 24 (13.3%)  CT: 84 (46.7%) | CC: 75 (44.1%)  TT: 19 (11.2%)  CT: 76 (44.7%) | 350 | p = .685 |
| rs706819^c^ | G: 282 (78.3%)  A: 78 (21.7%) | G: 260 (76.5%)  A: 80 (23.5%) | 700 | p = .554 | GG: 111 (61.7%)  AA: 9 (5.0%)  GA: 60 (33.3%) | GG: 98 (57.6%)  AA: 8 (4.7%)  GA: 64 (37.6%) | 350 | p = .444 |
| *TGFB1* |  |  |  |  |  |  |  |  |
| rs4803455 | C: 176 (48.9%)  A: 184 (51.1%) | C: 183 (53.8%)  A: 157 (46.2%) | 700 | p = .192 | CC: 41 (22.8%)  AA: 45 (25.0%)  CA: 94 (52.2%) | CC: 52 (30.6%)  AA: 39 (22.9%)  CA: 79 (46.5%) | 350 | p = .253 |
| rs1800469^c^ | C: 259 (71.9%)  T: 101 (28.1%) | C: 230 (67.6%)  T: 110 (32.4%) | 700 | p = .216 | CC: 94 (52.2%)  TT: 15 (8.3%)  CT: 71 (48.3%) | CC: 81 (47.6%)  TT: 21 (12.4%)  CT: 68 (40.0%) | 350 | p = .392 |
| rs1800468^c^ | C: 325 (90.8%)  T: 33 (9.2%) | C: 320 (92.0%)  T: 28 (8.0%) | 706 | p = .578 | CC: 147 (82.1%)  TT: 1 (.6%)  CT: 31 (17.3%) | CC: 149 (85.6%)  TT: 3 (1.7%)  CT: 22 (12.6%) | 353 | p = .370 |
| rs4803457 | C: 229 (63.6%)  T: 131 (36.4%) | C: 202 (59.4%)  T: 138 (40.6%) | 700 | p = .254 | CC: 71 (39.4%)  TT: 22 (12.2%)  CT: 87 (48.3%) | CC: 64 (37.6%)  TT: 32 (18.8%)  CT: 74 (43.5%) | 350 | p = .225 |
| *ENG* |  |  |  |  |  |  |  |  |
| rs10987746 | T: 192 (53.6%)  C: 166 (46.4%) | T: 162 (47.6%)  C: 178 (52.4%) | 698 | p = .114 | TT: 51 (28.5%)  CC: 38 (21.2%)  TC: 90 (50.3%) | TT: 38 (22.4%)  CC: 46 (27.1%)  TC: 86 (50.6%) | 349 | p = .283 |
| rs10819309 | G: 219 (61.2%)  A: 139 (38.8%) | G: 223 (65.6%)  A: 117 (34.4%) | 698 | p = .227 | GG: 65 (36.3%)  AA: 25 (14.0%)  GA: 89 (49.7%) | GG: 75 (44.1%)  AA: 22 (12.9%)  GA: 73 (42.9%) | 349 | p = .324 |
| rs10760505 | C: 222 (62.0%)  T: 136 (38.0%) | C: 205 (60.7%)  T: 133 (39.3%) | 696 | p = .708 | CC: 70 (39.1%)  TT: 27 (15.1%)  CT: 82 (45.8%) | CC: 64 (37.9%)  TT: 28 (16.6%)  CT: 77 (45.6%) | 348 | p = .925 |
| rs11792480 | G: 258 (71.7%)  A: 102 (28.3%) | G: 214 (63.0%)  A: 126 (37.0%) | 700 | **p = .014** | GG: 96 (53.3%)  AA: 18 (10.0%)  AG: 66 (36.7%) | GG: 74 (43.5%)  AA: 30 (17.6%)  AG: 66 (38.8%) | 350 | p = .062 |
| rs10121110 | A: 235 (66.0%)  G: 121 (34.0%) | A: 188 (55.3%)  G: 152 (44.7%) | 696 | **p = .004** | AA: 81 (45.5%)  GG: 24 (13.5%)  AG: 73 (41.0%) | AA: 56 (32.9%)  GG: 38 (22.4%)  AG: 76 (44.7%) | 348 | **p = .022** |
| *TGFBR2* |  |  |  |  |  |  |  |  |
| rs6550005^c^ | G: 304 (84.0%)  A: 58 (16.0%) | G: 270 (77.6%)  A: 78 (22.4%) | 710 | **p = .031** | GG: 130 (71.8%)  AA: 7 (3.9%)  GA: 44 (24.3%) | GG: 107 (61.5%)  AA: 11 (6.3%)  GA: 56 (32.2%) | 355 | **p = .039** |
| rs11129420 | A: 193 (53.3%)  T: 169 (46.7%) | A: 180 (51.7%)  T: 168 (48.3%) | 710 | p = .671 | AA: 50 (27.6%)  TT: 38 (21.0%)  TA: 93 (51.4%) | AA: 46 (26.4%)  TT: 40 (23.0%)  TA: 88 (50.6%) | 355 | p = .897 |
| rs6802220 | G: 216 (59.7%)  A: 146 (40.3%) | G: 200 (57.5%)  A: 148 (42.5%) | 710 | p = .554 | GG: 64 (35.4%)  AA: 29 (16.0%)  AG: 88 (48.6%) | GG: 52 (29.9%)  AA: 26 (14.9%)  AG: 96 (55.2%) | 355 | p = .446 |
| rs17025785 | T: 240 (66.3%)  C: 122 (33.7%) | T: 233 (67.0%)  C: 115 (33.0%) | 710 | p = .862 | TT: 79 (43.6%)  CC: 20 (11.0%)  TC: 82 (45.3%) | TT: 77 (44.3%)  CC: 18 (10.3%)  TC: 79 (45.4%) | 355 | p = .976 |
| rs4522809 | C: 185 (51.1%)  T: 177 (48.9%) | C: 171 (49.1%)  T: 177 (50.9%) | 710 | p = .603 | CC: 48 (26.5%)  TT: 44 (24.3%)  CT: 89 (49.2%) | CC: 43 (24.7%)  TT: 46 (26.4%)  CT: 85 (48.8%) | 355 | p = .872 |
| rs4955212^c^ | C: 257 (71.8%)  T: 101 (28.2%) | C: 257 (74.7%)  T: 87 (25.3%) | 702 | p = .383 | CC: 91 (50.8%)  TT: 13 (7.3%)  CT: 75 (41.9%) | CC: 96 (55.8%)  TT: 11 (6.4%)  CT: 65 (37.8%) | 351 | p = .350 |
| rs5020833^c^ | C: 252 (69.6%)  G: 110 (30.4%) | C: 242 (69.5%)  G: 106 (30.5%) | 710 | p > .999 | CC: 88 (48.6%)  GG: 17 (9.4%)  CG: 76 (42.0%) | CC: 83 (47.7%)  GG: 15 (8.6%)  CG: 76 (43.7%) | 355 | p = .863 |
| rs6809777^c^ | C: 270 (74.6%)  T: 92 (25.4%) | C: 255 (73.3%)  T: 93 (26.7%) | 710 | p = .689 | CC: 103 (56.9%)  TT: 14 (7.7%)  CT: 64 (35.4%) | CC: 97 (55.7%)  TT: 16 (9.2%)  CT: 61 (35.1%) | 355 | p = .826 |
| rs12487185^c^ | A: 261 (72.1%)  G: 101 (27.9%) | A: 248 (71.3%)  G: 101 (28.7%) | 710 | p = .806 | AA: 101 (55.8%)  GG: 21 (11.6%)  GA: 59 (32.6%) | AA: 87 (50.0%)  GG: 13 (7.5%)  GA: 74 (42.5%) | 355 | p = .274 |
| rs11924422 | A: 209 (57.7%)  C: 153 (42.3%) | A: 195 (56.0%)  C: 153 (44.0%) | 710 | p = .647 | AA: 60 (33.1%)  CC: 32 (17.7%)  CA: 89 (49.2%) | AA: 57 (32.8%)  CC: 36 (20.7%)  CA: 81 (46.6%) | 355 | p = .759 |
| rs13083813 | T: 221 (61.4%)  A: 139 (38.6%) | T: 220 (63.2%)  A: 128 (36.8%) | 708 | p = .617 | TT: 68 (37.8%)  AA: 27 (15.0%)  AT: 85 (47.2%) | TT: 70 (40.2%)  AA: 24 (13.8%)  AT: 80 (46.0%) | 354 | p = .880 |
| rs13075948^c^ | C: 261 (72.1%)  T: 101 (27.9%) | C: 254 (73.0%)  T: 94 (27.0%) | 710 | p = .791 | CC: 97 (53.6%)  TT: 17 (9.4%)  CT: 67 (37.0%) | CC: 90 (51.7%)  TT: 10 (5.7%)  CT: 74 (42.5%) | 355 | p = .725 |
| rs1155708 | G: 247 (68.2%)  A: 115 (31.8%) | G: 233 (67.3%)  A: 113 (32.7%) | 708 | p = .806 | GG: 87 (48.1%)  AA: 21 (11.6%)  GA: 73 (40.3%) | GG: 79 (45.7%)  AA: 19 (11.0%)  GA: 75 (43.4%) | 354 | p = .847 |
| rs13086588 | T: 246 (68.0%)  G: 116 (32.0%) | T: 241 (69.3%)  G: 107 (30.7%) | 710 | p = .708 | TT: 87 (48.1%)  GG: 22 (12.2%)  GT: 72 (39.8%) | TT: 86 (49.4%)  GG: 19 (10.9%)  GT: 69 (39.7%) | 355 | p = .927 |
| rs2082224^c^ | G: 278 (76.8%)  A: 84 (23.2%) | G: 265 (76.1%)  A: 83 (23.9%) | 710 | p = .841 | GG: 108 (59.7%)  AA: 11 (6.1%)  GA: 62 (34.3%) | GG: 100 (57.5%)  AA: 9 (5.2%)  GA: 65 (37.4%) | 355 | p = .674 |
| rs1078985^c^ | T: 271 (75.7%)  C: 87 (24.3%) | T: 248 (72.9%)  C: 92 (27.1%) | 698 | p = .403 | TT: 106 (59.2%)  CC: 14 (7.8%)  TC: 59 (33.0%) | TT: 88 (51.8%)  CC: 10 (5.9%)  TC: 72 (42.4%) | 349 | p = .161 |
| rs1036097 | G: 188 (51.9%)  A: 174 (48.1%) | G: 187 (53.7%)  A: 161 (46.3%) | 710 | p = .632 | GG: 49 (27.1%)  AA: 42 (23.3%)  GA: 90 (49.7%) | GG: 52 (29.9%)  AA: 39 (22.4%)  GA: 83 (47.7%) | 355 | p = .841 |
| rs995435^c^ | C: 263 (73.5%)  T: 95 (26.5%) | C: 250 (74.0%)  T: 88 (26.0%) | 696 | p = .888 | CC: 99 (55.3%)  TT: 15 (8.4%)  CT: 65 (36.3%) | CC: 91 (53.8%)  TT: 10 (5.9%)  CT: 68 (40.2%) | 348 | p = .784 |
| rs6792117 | A: 185 (51.7%)  G: 173 (48.3%) | A: 181 (52.0%)  G: 167 (48.0%) | 706 | p = .920 | AA: 48 (26.8%)  GG: 42 (23.5%)  GA: 89 (49.7%) | AA: 45 (25.9%)  GG: 38 (21.8%)  GA: 91 (52.3%) | 353 | p = .883 |
| rs749794^c^ | T: 252 (70.0%)  C: 108 (30.0%) | T: 225 (66.2%)  C: 115 (33.8%) | 700 | p = .277 | TT: 90 (50.0%)  CC: 18 (10.0%)  TC: 72 (40.0%) | TT: 70 (41.2%)  CC: 15 (8.8%)  TC: 85 (50.0%) | 350 | p = .098 |
| rs3773640^c^ | A: 276 (76.2%)  T: 86 (23.8%) | A: 262 (75.3%)  T: 86 (24.7%) | 710 | p = .764 | AA: 109 (60.2%)  TT: 14 (7.7%)  AT: 58 (32.0%) | AA: 95 (54.6%)  TT: 7 (4.0%)  AT: 72 (41.4%) | 355 | p = .284 |
| rs3773644 | C: 220 (60.8%)  T: 142 (39.2%) | C: 220 (63.2%)  T: 128 (36.8%) | 710 | p = .502 | CC: 64 (35.4%)  TT: 25 (13.8%)  CT: 92 (50.8%) | CC: 69 (39.7%)  TT: 23 (13.2%)  CT: 82 (47.1%) | 355 | p = .702 |
| rs3773645^c^ | C: 253 (69.9%)  G: 109 (30.1%) | C: 242 (69.9%)  G: 104 (30.1%) | 708 | p > .999 | CC: 87 (48.1%)  GG: 15 (8.3%)  CG: 79 (43.6%) | CC: 82 (47.4%)  GG: 13 (7.5%)  CG: 78 (45.1%) | 354 | p = .900 |
| rs3773652 | A: 196 (54.1%)  G: 166 (45.9%) | A: 173 (49.7%)  G: 175 (50.3%) | 710 | p = .237 | AA: 57 (31.5%)  GG: 42 (23.2%)  AG: 82 (45.3%) | AA: 49 (28.2%)  GG: 50 (28.7%)  AG: 75 (43.1%) | 355 | p = .479 |
| rs2043136^c^ | T: 274 (75.7%)  C: 88 (24.3%) | T: 246 (70.7%)  C: 102 (29.3%) | 710 | p = .133 | TT: 106 (58.6%)  CC: 13 (7.2%)  TC: 62 (34.3%) | TT: 87 (50.0%)  CC: 15 (8.6%)  TC: 72 (41.4%) | 355 | p = .105 |
| rs1346907 | C: 194 (53.6%)  T: 168 (46.4%) | C: 180 (51.7%)  T: 168 (48.3%) | 710 | p = .617 | CC: 62 (34.3%)  TT: 49 (27.1%)  CT: 70 (38.7%) | CC: 52 (29.9%)  TT: 46 (26.4%)  CT: 76 (43.7%) | 355 | p = .582 |
| rs876688 | G: 239 (66.4%)  A: 121 (33.6%) | G: 228 (65.5%)  A: 120 (34.5%) | 708 | p = .806 | GG: 85 (47.2%)  AA: 26 (14.4%)  GA: 69 (38.3%) | GG: 74 (42.5%)  AA: 20 (11.5%)  GA: 80 (46.0%) | 354 | p = .324 |
| rs877572 | G: 193 (53.3%)  C: 169 (46.7%) | G: 186 (53.4%)  C: 162 (46.6%) | 710 | p > .009 | GG: 59 (32.6%)  CC: 47 (26.0%)  CG: 75 (41.4%) | GG: 53 (30.5%)  CC: 41 (23.6%)  CG: 80 (46.0%) | 355 | p = .686 |
| rs9843942 | G: 218 (60.6%)  A: 142 (39.4%) | G: 221 (63.5%)  A: 127 (36.5%) | 708 | p = .420 | GG: 68 (37.8%)  AA: 30 (16.7%)  GA: 82 (45.6%) | GG: 69 (39.7%)  AA: 22 (12.6%)  GA: 83 (47.7%) | 354 | p = .565 |
| rs3773663 | G: 219 (60.5%)  A: 143 (39.5%) | G: 210 (60.3%)  A: 138 (39.7%) | 710 | p > .999 | GG: 69 (38.1%)  AA: 31 (17.1%)  AG: 81 (44.8%) | GG: 62 (35.6%)  AA: 26 (14.9%)  AG: 86 (49.4%) | 355 | p = .662 |
| rs744751^c^ | C: 269 (74.3%)  T: 93 (25.7%) | C: 253 (72.7%)  T: 95 (27.3%) | 710 | p = .624 | CC: 105 (58.0%)  TT: 17 (9.4%)  TC: 59 (32.6%) | CC: 90 (51.7%)  TT: 11 (6.3%)  TC: 73 (42.0%) | 355 | p = .234 |

^a^ *χ2* test of independence testing association between allele and preeclampsia status

^b^ *χ2* test of independence testing association between SNP genotype (homozygote wildtype, homozygote variant, heterozygote) and preeclampsia status

^c^ SNP genotypes dichotomized (homozygote wildtype, homozygote variant + heterozygote) due to small homozygote variant frequencies in either cases, controls, or both

**Table S5. Results of association analysis in black subgroup (Total N=60)**

| Gene  SNP | Allele Counts (%) Cases | Allele Counts (%) Controls | Total Alleles (*n)* | Allele Test^a^ | Genotype Counts (%) Cases | Genotype Counts (%) Controls | Total Genotypes (*n*) | Genotype Test^b^ |
| --- | --- | --- | --- | --- | --- | --- | --- | --- |
| *ALK5 (TGFBR1)* |  |  |  |  |  |  |  |  |
| rs6478974^c^ | T: 40 (69.0%)  A: 18 (31.0%) | T: 50 (83.3%)  A: 10 (16.7%) | 118 | p = .067 | TT: 15 (51.7%)  AA: 4 (13.8%)  TA: 10 (34.5%) | TT: 21 (70.0%)  AA: 1 (3.3%)  TA: 8 (26.7%) | 59 | p = .150 |
| rs10739778 | A: 46 (79.3%)  C: 12 (20.7%) | A: 34 (56.7%)  C: 26 (43.3%) | 118 | **p = .008** | AA: 19 (65.5%)  CC: 2 (6.9%)  AC: 8 (27.6%) | AA: 9 (30.0%)  CC: 5 (16.7%)  AC: 16 (53.3%) | 59 | **p = .028** |
| rs420549^c^ | G: 53 (91.4%)  C: 5 (8.6%) | G: 51 (85.0%)  C: 9 (15.0%) | 118 | p = .284 | GG: 24 (82.8%)  CC: 0 (0.0%)  GC: 5 (17.2%) | GG: 22 (73.3%)  CC: 1 (3.3%)  GC: 7 (23.3%) | 59 | p = .383 |
| *ALK1* |  |  |  |  |  |  |  |  |
| rs3759178 | T: 33 (56.9%)  G: 25 (43.1%) | T: 38 (63.3%)  G: 22 (36.7%) | 118 | p = .475 | TT: 10 (34.5%)  GG: 6 (20.7%)  GT: 13 (44.8%) | TT: 14 (46.7%)  GG: 6 (20.0%)  GT: 10 (33.3%) | 59 | p = .594 |
| rs11169953 | C: 34 (58.6%)  T: 24 (41.4%) | C: 29 (48.3%)  T: 31 (51.7%) | 118 | p = .264 | CC: 12 (41.4%)  TT: 7 (24.1%)  CT: 10 (34.5%) | CC: 9 (30.0%)  TT: 10 (33.3%)  CT: 11 (36.7%) | 59 | p = .610 |
| rs706819^c^ | G: 43 (74.1%)  A: 15 (25.9%) | G: 40 (66.7%)  A: 20 (33.3%) | 118 | p = .374 | GG: 16 (55.2%)  AA: 2 (6.9%)  GA: 11 (37.9%) | GG: 13 (43.3%)  AA: 3 (10.0%)  GA: 14 (46.7%) | 59 | p = .363 |
| *TGFB1* |  |  |  |  |  |  |  |  |
| rs4803455 | C: 33 (56.9%)  A: 25 (43.1%) | C: 27 (45.0%)  A: 33 (55.0%) | 118 | p = .196 | CC: 8 (27.6%)  AA: 4 (13.8%)  CA: 17 (58.6%) | CC: 10 (33.3%)  AA: 13 (43.3%)  CA: 7 (23.3%) | 59 | **p = .010** |
| rs1800469^c^ | C: 40 (69.0%)  T: 18 (31.0%) | C: 46 (76.7%)  T: 14 (23.3%) | 118 | p = .348 | CC: 13 (44.8%)  TT: 2 (6.9%)  CT: 14 (48.3%) | CC: 19 (63.3%)  TT: 3 (10.0%)  CT: 8 (26.7%) | 59 | p = .154 |
| rs1800468^c^ | C: 57 (95.0%)  T: 3 (5.0%) | C: 57 (95.0%)  T: 3 (5.0%) | 120 | p > .999 | CC: 27 (90.0%)  TT: 0 (0.0%)  CT: 3 (10.0%) | CC: 27 (90.0%)  TT: 0 (0.0%)  CT: 3 (10.0%) | 60 | p > .999 |
| rs4803457 | C: 25 (43.1%)  T: 33 (56.9%) | C: 36 (60.0%)  T: 24 (40.0%) | 118 | p = .066 | CC: 4 (13.8%)  TT: 8 (27.6%)  CT: 17 (58.6%) | CC: 14 (46.7%)  TT: 8 (26.7%)  CT: 8 (26.7%) | 59 | **p = .012** |
| *ENG* |  |  |  |  |  |  |  |  |
| rs10987746 | T: 31 (53.4%)  C: 27 (46.6%) | T: 33 (55.0%)  C: 27 (45.0%) | 118 | p = .862 | TT: 9 (31.0%)  CC: 7 (24.1%)  TC: 13 (44.8%) | TT: 10 (33.3%)  CC: 7 (23.3%)  TC: 13 (43.3) | 59 | p = .982 |
| rs10819309 | G: 41 (70.7%)  A: 17 (29.3%) | G: 39 (65.0%)  A: 21 (35%) | 118 | p = .507 | GG: 16 (55.2%)  AA: 4 (13.8%)  GA: 9 (31.0%) | GG: 13 (43.3%)  AA: 4 (13.3%)  GA: 13 (43.3%) | 59 | p = .590 |
| rs10760505^c^ | C: 50 (86.2%)  T: 8 (13.8%) | C: 54 (90.0%)  T: 6 (10.0%) | 118 | p = .522 | CC: 22 (75.9%)  TT: 1 (3.4%)  CT: 6 (20.7%) | CC: 25 (83.3%)  TT: 1 (3.3%)  CT: 4 (13.3%) | 59 | p = .476 |
| rs11792480^c^ | G: 49 (84.5%)  A: 9 (15.5%) | G: 55 (91.7%)  A: 5 (8.3%) | 118 | p = .227 | GG: 22 (75.9%)  AA: 2 (6.9%)  AG: 5 (17.2%) | GG: 25 (83.3%)  AA: 0 (0.0%)  AG: 5 (16.7%) | 59 | p = .476 |
| rs10121110 | G: 37 (63.8%)  A: 21 (36.2%) | G: 36 (60.0%)  A: 24 (40.0%) | 118 | p = .671 | GG: 13 (44.8%)  AA: 5 (17.2%)  AG: 11 (37.9%) | GG: 10 (33.3%)  AA: 4 (13.3%)  AG: 16 (53.3%) | 59 | p = .472 |
| *TGFBR2* |  |  |  |  |  |  |  |  |
| rs6550005 | G: 32 (53.3%)  A: 28 (46.7%) | G: 44 (73.3%)  A: 16 (26.7%) | 120 | **p = .023** | GG: 9 (30.0%)  AA: 7 (23.3%)  GA: 14 (46.7%) | GG: 17 (56.7%)  AA: 3 (10.0%)  GA: 10 (33.3%) | 60 | p = .094 |
| rs11129420 | A: 40 (66.7%)  T: 20 (33.3%) | A: 45 (75.0%)  T: 15 (25.0%) | 120 | p = .315 | AA: 13 (43.3%)  TT: 3 (10.0%)  TA: 14 (46.7%) | AA: 18 (60.0%)  TT: 3 (10.0%)  TA: 9 (30.0%) | 60 | p = .419 |
| rs6802220 | A: 37 (61.7%)  G: 23 (38.3%) | A: 40 (66.7%)  G: 20 (33.3%) | 120 | p = .566 | AA: 11 (36.7%)  GG: 4 (13.3%)  AG: 15 (50.0%) | AA: 15 (50.0%)  GG: 5 (16.7%)  AG: 10 (33.3%) | 60 | p = .418 |
| rs17025785^c^ | T: 42 (70.0%)  C: 18 (30.0%) | T: 42 (70.0%)  C: 18 (30.0%) | 120 | p > .999 | TT: 14 (46.7%)  CC: 2 (6.7%)  TC: 14 (46.7%) | TT: 14 (46.7%)  CC: 2 (6.7%)  TC: 14 (46.7%) | 60 | p > .999 |
| rs4522809 | T: 33 (55.0%)  C: 27 (45.0%) | T: 30 (50.0%)  C: 30 (50.0%) | 120 | p = .584 | TT: 8 (26.7%)  CC: 5 (16.7%)  CT: 17 (56.7%) | TT: 5 (16.7%)  CC: 5 (16.7%)  CT: 20 (66.7%) | 60 | p = .626 |
| rs4955212^c^ | C: 48 (80.0%)  T: 12 (20.0%) | C: 47 (78.3%)  T: 13 (21.7%) | 120 | p = .823 | CC: 18 (60.0%)  TT: 0 (0%)  CT: 12 (40.0%) | CC: 18 (60.0%)  TT: 1 (3.3%)  CT: 11 (36.7%) | 60 | p > .999 |
| rs5020833^c^ | C: 45 (75.0%)  G: 15 (25.0%) | C: 40 (66.7%)  G: 20 (33.3%) | 120 | p = .315 | GG: 17 (56.7%)  AA: 2 (6.7%)  GA: 11 (36.7%) | GG: 15 (50.0%)  AA: 5 (16.7%)  GA: 10 (33.3%) | 60 | p = .605 |
| rs6809777^c^ | C: 48 (80.0%)  T: 12 (20.0%) | C: 45 (75.0%)  T: 15 (25.0%) | 120 | p = .512 | CC: 21 (70.0%)  TT: 3 (10.0%)  CT: 6 (20.0%) | CC: 17 (56.7%)  TT: 2 (6.7%)  CT: 11 (36.7%) | 60 | p = .422 |
| rs12487185^c^ | A: 51 (85.0%)  G: 9 (15.0%) | A: 45 (75.0%)  G: 15 (25.0%) | 120 | p = .170 | AA: 23 (76.7%)  GG: 2 (6.7%)  GA: 5 (16.7%) | AA: 19 (63.3%)  GG: 4 (13.3%)  GA: 7 (23.3%) | 60 | p = .260 |
| rs11924422^c^ | A: 33 (55.0%)  C: 27 (45.0%) | A: 43 (71.7%)  C: 17 (28.3%) | 120 | p = .058 | AA: 8 (26.7%)  CC: 5 (16.7%)  CA: 17 (56.7%) | AA: 15 (50.0%)  CC: 2 (6.7%)  CA: 13 (43.3%) | 60 | p = .063 |
| rs13083813^c^ | T: 44 (73.3%)  A: 16 (26.7%) | T: 49 (81.7%)  A: 11 (18.3%) | 120 | p = .275 | TT: 15 (50.0%)  AA: 1 (3.3%)  AT: 14 (46.7%) | TT: 19 (63.3%)  AA: 0 (0.0%)  AT: 11 (36.7%) | 60 | p = .297 |
| rs13075948^c^ | C: 48 (70.0%)  T: 12 (30.0%) | C: 49 (81.7%)  T: 11 (18.3%) | 120 | p = .823 | CC: 19 (63.3%)  TT: 1 (3.3%)  CT: 10 (33.3%) | CC: 20 (66.7%)  TT: 1 (3.3%)  CT: 9 (30.0%) | 60 | p = .787 |
| rs1155708 | G: 39 (65.0%)  A: 21 (35.0%) | G: 34 (56.7%)  A: 26 (43.3%) | 120 | p = .351 | GG: 12 (40.0%)  AA: 3 (10.0%)  GA: 15 (50.0%) | GG: 10 (33.3%)  AA: 6 (20.0%)  GA: 14 (46.7%) | 60 | p = .611 |
| rs13086588 | T: 41 (68.3%)  G: 19 (31.7%) | T: 34 (56.7%)  G: 26 (43.3%) | 120 | p = .187 | TT: 14 (46.7%)  GG: 3 (10.0%)  GT: 13 (43.3%) | TT: 11 (36.7%)  GG: 7 (23.3%)  GT: 12 (40.0%) | 60 | p = .368 |
| rs2082224^c^ | G: 46 (76.7%)  A: 14 (23.3%) | G: 42 (70.0%)  A: 18 (30.0%) | 120 | p = .410 | GG: 17 (56.7%)  AA: 1 (3.3%)  GA: 12 (40.0%) | GG: 15 (50.0%)  AA: 3 (10.0%)  GA: 12 (40.0%) | 60 | p = .605 |
| rs1078985^c^ | T: 50 (83.3%)  C: 10 (16.7%) | T: 48 (82.8%)  C: 10 (17.2%) | 118 | p = .920 | TT: 22 (73.3%)  CC: 2 (6.7%)  TC: 6 (20.0%) | TT: 21 (72.4%)  CC: 2 (6.9%)  TC: 6 (20.7%) | 59 | p = .937 |
| rs1036097^c^ | G: 39 (65.0%)  A: 21 (35.0%) | G: 41 (68.3%)  A: 19 (31.7%) | 120 | p = .699 | GG: 11 (36.7%)  AA: 2 (6.7%)  GA: 17 (56.7%) | GG: 16 (53.3%)  AA: 5 (16.7%)  GA: 9 (30.0%) | 60 | p = .194 |
| rs995435 | C: 40 (66.7%)  T: 20 (33.3%) | C: 36 (62.1%)  T: 22 (37.9%) | 118 | p = .603 | CC: 14 (46.7%)  TT: 4 (13.3%)  CT: 12 (40.0%) | CC: 12 (41.4%)  TT: 5 (17.2%)  CT: 12 (41.4%) | 59 | p = .940 |
| rs6792117 | A: 35 (58.3%)  G: 25 (41.7%) | A: 39 (65.0%)  G: 21 (35.0%) | 120 | p = .454 | AA: 8 (26.7%)  GG: 3 (10.0%)  GA: 19 (63.3%) | AA: 14 (46.7%)  GG: 5 (16.7%)  GA: 11 (36.7%) | 60 | p = .140 |
| rs749794^c^ | T: 41 (70.7%)  C: 17 (29.3%) | T: 32 (53.3%)  C: 28 (46.7%) | 118 | p = .052 | TT: 14 (48.3%)  CC: 2 (6.9%)  TC: 13 (44.8%) | TT: 10 (33.3%)  CC: 8 (26.7%)  TC: 12 (40.0%) | 59 | p = .243 |
| rs3773640^c^ | A: 47 (78.3%)  T: 13 (21.7%) | A: 43 (71.7%)  T: 17 (28.3%) | 120 | p = .399 | AA: 20 (66.7%)  TT: 3 (10.0%)  AT: 7 (23.3%) | AA: 15 (50.0%)  TT: 2 (6.7%)  AT: 13 (43.3%) | 60 | p = .190 |
| rs3773644^c^ | C: 37 (61.7%)  T: 23 (38.3%) | C: 44 (73.3%)  T: 16 (26.7%) | 120 | p=0.173 | CC: 11 (36.7%)  TT: 4 (13.3%)  CT: 15 (50.0%) | CC: 16 (53.3%)  TT: 2 (6.7%)  CT: 12 (40.0%) | 60 | p = .194 |
| rs3773645^c^ | C: 45 (75.0%)  G: 15 (25.0%) | C: 48 (70.0%)  G: 12 (30.0%) | 120 | p = .512 | CC: 20 (66.7%)  GG: 5 (16.7%)  CG: 5 (16.7%) | CC: 19 (63.3%)  GG: 1 (3.3%)  CG: 10 (33.3%) | 60 | p = .787 |
| rs3773652 | G: 30 (50.0%)  A: 30 (50.0%) | G: 34 (56.7%)  A: 26 (43.3%) | 120 | p = .462 | GG: 7 (23.3%)  AA: 7 (23.3%)  AG: 16 (53.3%) | GG: 8 (26.7%)  AA: 4 (13.3%)  AG: 18 (60.0%) | 60 | p = .606 |
| rs2043136^c^ | T: 48 (70.0%)  C: 12 (30.0%) | T: 45 (75.0%)  C: 15 (15.0%) | 120 | p = .512 | TT: 19 (63.3%)  CC: 1 (3.3%)  TC: 10 (33.3%) | TT: 16 (53.3%)  CC: 1 (3.3%)  TC: 13 (43.3%) | 60 | p = .432 |
| rs1346907^c^ | C: 48 (70.0%)  T: 12 (30.0%) | C: 38 (63.3%)  T: 22 (36.7%) | 120 | **p = .043** | CC: 18 (60.0%)  TT: 0 (0%)  CT: 12 (40.0%) | CC: 13 (43.3%)  TT: 5 (16.7%)  CT: 12 (40.0%) | 60 | p = .196 |
| rs876688 | G: 32 (53.3%)  A: 28 (46.7%) | G: 36 (60.0%)  A: 24 (40.0%) | 120 | p = .462 | GG: 7 (23.3%)  AA: 5 (16.7%)  GA: 18 (60.0%) | GG: 11 (36.7%)  AA: 5 (16.7%)  GA: 14 (46.7%) | 60 | p = .499 |
| rs877572^c^ | G: 48 (70.0%)  C: 12 (30.0%) | G: 37 (61.7%)  C: 23 (38.3%) | 120 | **p = .027** | GG: 18 (60.0%)  CC: 0 (0.0%)  CG: 12 (40.0%) | GG: 13 (43.3%)  CC: 6 (20.0%)  CG: 11 (36.7%) | 60 | p = .196 |
| rs9843942 | G: 35 (58.3%)  A: 25 (41.7%) | G: 29 (48.3%)  A: 31 (51.7%) | 120 | p = .271 | GG: 11 (36.7%)  AA: 6 (20.0%)  GA: 13 (43.3%) | GG: 8 (26.7%)  AA: 9 (30.0%)  GA: 13 (43.3%) | 60 | p = .585 |
| rs3773663 | G: 28 (46.7%)  A: 32 (53.3%) | G: 38 (63.3%)  A: 22 (36.7%) | 120 | p = .066 | GG: 6 (20.0%)  AA: 8 (26.7%)  AG: 16 (53.3%) | GG: 12 (40.0%)  AA: 4 (13.3%)  AG: 14 (46.7%) | 60 | p = .177 |
| rs744751^c^ | C: 50 (83.3%)  T: 10 (16.7%) | C: 55 (91.7%)  T: 5 (8.3%) | 120 | p = .168 | CC: 21 (70.0%)  TT: 1 (3.3%)  TC: 8 (26.7%) | CC: 25 (83.3%)  TT: 0 (0.0%)  TC: 5 (16.7%) | 60 | p = .222 |

^a^ *χ2* test of independence or Fisher’s exact test testing association between allele and preeclampsia status

^b^ *χ2* test of independence or Fisher’s Exact exact test testing association between SNP genotype (homozygote wildtype, homozygote variant, heterozygote) and preeclampsia status

^c^ SNP genotypes dichotomized (homozygote wildtype, homozygote variant + heterozygote) due to small homozygote variant frequencies in either cases, controls, or both
